# Using Eye-Tracking to Investigate an Activation-Based Account of False Hearing in Younger and Older Adults

**DOI:** 10.3389/fpsyg.2022.821044

**Published:** 2022-05-16

**Authors:** Eric Failes, Mitchell S. Sommers

**Affiliations:** Department of Psychological and Brain Sciences, Washington University in St. Louis, St. Louis, MO, United States

**Keywords:** false hearing, speech perception, aging, eye-tracking, inhibition

## Abstract

Several recent studies have demonstrated context-based, high-confidence misperceptions in hearing, referred to as *false hearing*. These studies have unanimously found that older adults are more susceptible to false hearing than are younger adults, which the authors have attributed to an age-related decline in the ability to inhibit the activation of a contextually predicted (but incorrect) response. However, no published work has investigated this activation-based account of false hearing. In the present study, younger and older adults listened to sentences in which the semantic context provided by the sentence was either unpredictive, highly predictive and valid, or highly predictive and misleading with relation to a sentence-final word in noise. Participants were tasked with clicking on one of four images to indicate which image depicted the sentence-final word in noise. We used eye-tracking to investigate how activation, as revealed in patterns of fixations, of different response options changed in real-time over the course of sentences. We found that both younger and older adults exhibited anticipatory activation of the target word when highly predictive contextual cues were available. When these contextual cues were misleading, younger adults were able to suppress the activation of the contextually predicted word to a greater extent than older adults. These findings are interpreted as evidence for an activation-based model of speech perception and for the role of inhibitory control in false hearing.

## Introduction

What a listener reports hearing is influenced by what they expect to hear. Indeed, there are many studies that demonstrate how speech perception is facilitated by the presence of valid semantic contexts, which allow the listener to anticipate what will be said (Hutchinson, 1989; Nittrouer and Boothroyd, 1990; Wingfield et al., 1991; Pichora-Fuller et al., 1995; Sommers and Danielson, 1999; Dubno et al., 2000; Benichov et al., 2012; Rogers et al., 2012; Sommers et al., 2015; Failes et al., 2020). The availability of contextual cues may be especially important when the speech signal is degraded—such as when speech is presented in noise or when the listener suffers from hearing loss—as the semantic cues may allow the listener to infer what was said in cases where acoustic information was missed.

A compelling demonstration of the influence of context in speech perception is *false hearing*, instances in which listeners erroneously report hearing a contextually predicted word when a similar sounding but unpredicted word is presented (Rogers et al., 2012; Sommers et al., 2015; Failes et al., 2020). For example, Sommers et al. (2015) had younger and older adults identify sentence-final words in three different conditions: (1) sentences providing no context for predicting the sentence-final word (baseline condition: *He was thinking about the sheep*); (2) sentences providing a valid context for predicting the sentence-final word (congruent condition: *The shepherd watched his sheep*); and (3) sentences in which the sentence-final word was a phonological neighbor of the predicted sentence-final word (incongruent condition: *The shepherd watched his sheath*). In all cases, the participant’s task was to report the sentence-final word, which was presented in background noise, and to judge their confidence in the accuracy of their response on a 0–100 point scale. To account for differences in hearing acuity across younger and older adults (see Morrell et al., 1996; Sommers et al., 2011 for hearing acuity trends across the lifespan), signal-to-noise ratios (SNRs) were set individually to obtain approximately 50% accuracy for all participants in the baseline condition. Importantly, all participants were warned that sentences would sometimes be misleading, and there were three times as many incongruent sentences as congruent sentences. Both the warning instructions and the disproportionate use of incongruent sentences should have discouraged a context-based response strategy. Despite conditions that discouraged using context, Sommers et al. found that both younger and older adults experienced false hearing in the incongruent condition (e.g., reported hearing *sheep* when presented *The shepherd watched his sheath*). However, older adults were more susceptible to and more confident in their false hearing responses than were younger adults (0.50 vs. 0.39). Older adults were also much more likely (0.16 vs. 0.04) than younger adults to report maximum confidence in cases of false hearing (100% confidence in the incorrect, but semantically predicted response). Participants’ continued use of contextual cues in conditions that discouraged use of context lends further support to the argument that both age groups—but especially older adults—relied on context as a basis for responding.

False hearing is often described as resulting from an inability to suppress an expected response, reflecting a failure of inhibitory control (Rogers et al., 2012; Sommers et al., 2015; Failes et al., 2020). Using the stimuli from Sommers et al. (2015) as an example, the sentence “*The shepherd watched his…*” creates a strong expectation of what word should follow (*sheep*). This expectation acts a source of increased activation of the expected lexical item. In the case of incongruent sentences, the participant must then suppress the highly activated word *sheep* to correctly hear the presented (but unpredicted) word *sheath*.

Although the role of inhibitory control in false hearing has yet to be tested directly, there is evidence that inhibitory control influences veridical speech perception. Sommers and Danielson (1999, Experiment 2), for example, found that better inhibitory control—as assessed by a composite of a selective attention paradigm developed by Garner (1974) and an auditory Stroop task (Stroop, 1935)—was associated with improved ability to identify lexically hard words (i.e., words with many, high-frequency phonological neighbors) but not lexically easy words (i.e., words with fewer, lower-frequency phonological neighbors) in noise. Additionally, the correlation between inhibitory control and the ability to identify lexically hard words was reduced when the target word was preceded by a high-predictability sentence (e.g., *She was walking along the path*) relative to when preceded by a low-predictability sentence (e.g., *She was thinking about the path*). The authors interpreted their findings within the Neighborhood Activation Model (NAM; Luce and Pisoni, 1998), which suggests that when listening to a spoken word, both the word and its phonological neighbors become activated in the mental lexicon and compete for perception. Sommers and Danielson suggested that inhibitory control might be used to suppress the activation of competing phonological neighbors, and that the availability of highly predictive (and valid) contextual cues may reduce the need to suppress competitors by selectively increasing the activation of the contextually congruent target word.

The proposal by Sommers and Danielson (1999, Experiment 2) that inhibitory control is needed to reduce activation of a target word’s phonological neighbors and that context increases the activation of semantically viable words is highly pertinent to the studies of false hearing. Recall that the target words in the incongruent condition of the study by Sommers et al. (2015) were phonological neighbors of the word predicted by context. Based on the NAM (Luce and Pisoni, 1998) and the findings of Sommers and Danielson, we would expect that hearing the incongruent target word (e.g., *sheath* in the sentence *The shepard watched his sheath*) would activate the contextually predicted phonological neighbor of the target word (e.g., *sheep*), and that the activation of this phonological neighbor would be boosted due to its compatibility with available contextual cues. This set of conditions should result in an increased probability that participants would mistakenly “hear” the contextually predicted phonological neighbor, a case of false hearing. Therefore, if the role of inhibitory control is to decrease activation of phonological neighbors of the target word as suggested by Sommers and Danielson, then we might expect that individuals with better inhibitory control would be less susceptible to false hearing than those with poorer inhibitory control. Given the well-established age-related decline in inhibitory control (Cohn et al., 1984; Hasher and Zacks, 1988; MacLeod, 1991; Hasher et al., 1997; Sommers and Danielson, 1999; Jacoby et al., 2005, Experiment 2; Sommers and Huff, 2003, Experiment 2; West and Alain, 2000), older adults’ increased susceptibility to false hearing may result, at least in part, from declines in the ability to inhibit activation on phonological neighbors activated by both semantic context and phonological similarity.

One experimental method that may be particularly useful for testing the inhibitory control account of false hearing is eye-tracking. Eye-tracking has been increasingly used to study language processing because it allows the researcher to observe changes in attentional focus over time, providing a real-time assessment of the processing that occurs before a response is made. In speech perception research, eye-tracking is often used within a visual world paradigm (Allopenna et al., 1998; Dahan and Gaskell, 2007; Revill and Spieler, 2012; Mishra and Singh, 2014; Ito et al., 2018; Kukona, 2020), in which different response options are depicted in the form of written words or pictures. It has been argued that changes in the proportion of fixations on the written words or pictures in the visual world paradigm can be used as an index of changes in activation, with more highly activated response options receiving a greater proportion of fixations than less highly activated options (Tanenhaus et al., 2000). Supporting this claim, images depicting high-frequency words, which are assumed to gain more activation than low-frequency words within speech perception models such as the NAM (Luce and Pisoni, 1998), tend to receive a greater proportion of fixations than images depicting low-frequency words (Dahan et al., 2001; Dahan and Gaskell, 2007; Revill and Spieler, 2012). Similarly, following from claim of Sommers and Danielson (1999) that words gain activation when supported by contextual cues, images depicting words that are congruent with available semantic context tend to receive a greater proportion of fixations than images depicting words that do not fit with context (Ito et al., 2018; Kukona, 2020). Therefore, the eye-tracking methodology could allow for a test of the inhibitory control account of false hearing. Misleading sentences, as in the incongruent condition of the study by Sommers et al. (2015), should lead to increased fixations on an image depicting the contextually predicted (but incorrect) word. To the extent that participants are able to suppress activation of the contextually predicted word when the unpredicted target word is presented, they should be able to fixate more on the image depicting the correct, but not predicted, target item.

Two recent studies (Ayasse and Wingfield, 2020; Harel-Arbeli et al., 2021) used eye-tracking to compare activation of semantic, rather than phonological, competitors in older and younger adults. Ayasse and Wingfield (2020) compared the time-course of gaze fixations for older and younger adults on sentence-final target items when a semantically plausible competitor was either present or absent as well as for a control condition in which context was not predictive of the sentence final item. For young adults, growth curve analyses indicated similar slopes of gaze fixations whether or not a semantically related competitor was presented. In contrast, older adults showed shallower slopes for target fixations (i.e., slower time-course of target fixations) when a semantic competitor was present compared to when an unrelated word was used as the response alternative. Moreover, the age-related difference in target fixations in the presence of a semantic competitor was driven in part by individual differences in inhibitory control as evidenced by greater interference from a semantic competitor for older adults with lower versus higher measures of inhibitory control. Similar results were reported by Harel-Arbeli et al. (2021) who found that older adults were slower than younger listeners to look at a target picture when a semantic competitor was present, suggesting an age-related decline in the ability to inhibit semantic competition.

In the present study, we used eye-tracking with the visual world paradigm to examine age-related changes in inhibiting phonological competition as revealed by fixations in a false hearing paradigm. Specifically, the current study investigated how activation of different response options change over the course of neutral, valid, and misleading sentence contexts. The visual world task was similar to the one used by Harel-Arbeli et al. (2021) but used the speech perception in noise (SPIN) task described earlier (Sommers et al., 2015) in which context preceding a sentence-final word in noise was either non-predictive (baseline condition), predictive and valid (congruent condition) or predictive but invalid (incongruent condition). As each sentence was played, four pictures were presented on the computer screen. The pictures depicted the target word (e.g., box), a phonological neighbor of the target word (e.g., fox), and two words that did not sound like the target word and were not predicted by the sentence context (e.g., key and paw). Using eye-tracking, we were able to determine how the proportion of fixations on each of the images changed over the course of the sentence and after the target word was presented.

We formed specific hypotheses regarding how younger and older adults’ fixation patterns would change over the course of baseline, congruent, and incongruent sentences. In the baseline condition, we predicted that the proportion of fixations on each of the images should remain approximately equal until the target word was presented since there were no contextual cues upon which to base an expectation. After the target word was presented in the baseline condition, fixations on the target image should increase in accordance with participants’ ability to accurately hear the target word. In congruent and incongruent sentences, we hypothesized that both younger and older adults would become increasingly fixated on the contextually predicted image leading up to presentation of the target word, demonstrating increasing anticipatory activation of the word supported by context. We predicted that this increased focus on the contextually predicted image might be greater for older than younger adults, reflecting older adults’ increased context-based responding demonstrated in previous studies (Rogers et al., 2012; Sommers et al., 2015; Failes et al., 2020). Whereas fixations on the target image should continue to increase for both age groups once the target word was presented in the congruent condition, we predicted that the age groups would differ in their reaction to presentation of the target word in the incongruent condition. Specifically, we predicted that younger adults would decrease their proportion of fixations on the contextually predicted (but not presented) visual image and increase their fixations on the unpredicted (but correct) target image after the target word was presented, reflecting their ability to suppress the activation of the expected word. Older adults, on the other hand, were expected to maintain or even increase their fixations on the contextually predicted image after the target word was presented in incongruent sentences, reflecting an inability to suppress the activation of the expected word. This would align with the theory that false hearing reflects a failure to inhibit a highly activated response and the findings of previous studies suggesting that older adults have poorer inhibitory control than younger adults (Cohn et al., 1984; Hasher and Zacks, 1988; MacLeod, 1991; Hasher et al., 1997; Sommers and Danielson, 1999; Jacoby et al., 2005, Experiment 2; Sommers and Huff, 2003, Experiment 2; West and Alain, 2000).

## Materials and Methods

### Participants

Participants were 23 younger adults ages 18–29 (*M* = 21.0, SD = 2.68) and 19 older adults ages 66–81 (*M* = 73.31, SD = 4.45). All participants were native English speakers who did not require the use of a hearing aid and self-reported normal or corrected-to-normal vision. To assess English language competency, participants completed the Shipley Vocabulary Test (Shipley, 1940) in which participants decided which of four words was most similar in meaning to 40 distinct target words. Good vocabulary knowledge was exhibited by both younger (*M* = 33.04, SD = 3.04) and older adults (*M* = 34.42, SD = 3.91), and the two groups did not differ in vocabulary knowledge, *t*(33.61) = −1.26, *p* > 0.05. Hearing thresholds were assessed for octave frequencies from 250 to 8,000 Hz in a sound-attenuating booth using standard audiometry. As would be expected due to age-related hearing loss (Morrell et al., 1996; Sommers et al., 2011), older adults (*M* = 23.68, SD = 11.78) had poorer best-ear PTAs (500/1,000/2,000 Hz frequencies) than younger adults (*M* = 4.42, SD = 3.39), *t*(20.47) = −6.90, *p* < 0.001.

### Stimuli and Materials

Stimuli in the SPIN task were 31 carrier sentences (baseline sentences: *The word is* *page*) and 62 high-predictability sentences (congruent sentences: *She put the toys in a* *box*) selected from the Revised Speech Perception in Noise Test (Bilger et al., 1984) or created specifically for this study. For half of the congruent sentences, the first or last sound in the sentence-final word was changed to form an alternative word that was not predicted by the sentence context (incongruent sentences: *She put the toys in the fox*). For half of these changed items, the onset was altered and for the remaining half we changed the offset. The length, frequency, phonological neighborhood density, and concreteness of target words were collected from the English Lexicon Project (Balota et al., 2007), and averages across sentence conditions are presented in Table 1. Target words did not differ significantly in terms of any of these lexical characteristics across sentence conditions, all *p*s > 0.05.

**Table 1:** Means and standard deviations of lexical characteristics of target words across conditions.

	Baseline		Congruent		Incongruent	
	*M*	SD	*M*	SD	*M*	SD
Length	4.20	0.76	4.37	0.85	4.23	1.01
Frequency	33,266.53	77,434.77	28,985.50	44,531.67	32,123.90	44,336.55
Phono N	19.07	9.45	18.67	9.89	20.80	11.16
Concreteness	4.66	0.26	4.71	0.41	4.61	0.39

*Phono N, Phonological neighborhood density*.

All sentences were recorded at 44,100 Hz and 16-bit resolution in a double-walled, sound-attenuating booth, and were spoken at a normal rate by a male with a Midwestern American accent. Periods of silence of different lengths were inserted at the start of each sentence so that the onset of the target word began at the same time on each trial to facilitate eye-tracking analyses. All sentences were played at an average amplitude of 64 dB sound pressure level (SPL).

Four images were gathered for each sentence for use in the visual world task: one depicting the target word (e.g., *box* for the sentence *She put the toys in the box*), one depicting a semantically unrelated phonological neighbor of the target word that acted as the target word in incongruent sentences (alternative image: *fox*), and two semantically unrelated foil words that did not sound like the target word (e.g., *key* and *paw*). Two foil images were included on each trial as opposed to including a second phonological neighbor or a semantic competitor of the target word so that participants would not divide their fixations across two images tapping the same source of information (semantic or phonological). This allowed for clearer assessment of how fixations were impacted by semantic congruency and phonological similarity. Each image was resized to 300 × 300 pixels. For images that did not have equal width and height, a white border was added to the shorter dimension to achieve the 300 × 300 size.

A pilot test was conducted to ensure that all images to be used in the visual world task were identifiable as the words they were meant to depict. Twenty younger adults participated in this pilot study. First, participants completed a study phase in which they saw each image along with the word the image was meant to depict for 2,000 ms. Participants then completed a test phase wherein each written word was presented at the center of the screen one at a time along with four images. One of the images depicted the written word at the center of the screen, and the other three images were the images paired with the initial image on trials in the SPIN task. For example, when the target word was fox, the four images on screen depicted a fox, a box, a key, and a paw, and the same four images were presented when the target word was box, key, and paw. The images were randomly assigned to one of the four quadrants of the screen. Participants were tasked with clicking on the image that depicted the word at the center of the screen. Average accuracy for identifying the image depicting each target word was 99.42%. In fact, only two images were identified correctly in less than 90% of cases, one of which was only used in the practice trials of the SPIN task (*joker*, Accuracy = 75%) and the other was a foil (*till*, Accuracy = 70%). Given the high identification accuracy of virtually all images in the pilot study and the similarity of the pilot study’s procedure to that of the SPIN task, we felt confident that participants would associate each image with the word they were intended to depict in the SPIN task.

A second pilot test was conducted to determine the SNR that would be needed for younger and older adults to achieve approximately 50% accuracy in the baseline condition of the SPIN task. We first tested younger adults at −4 dB SPL and older adults at +1 dB SPL, SNRs used in a similar SPIN task in a prior study (Failes et al., 2020). However, accuracy was at ceiling in the baseline condition for younger adults using the −4 dB SPL SNR (*M* = 0.93), and older adults’ performance at the +1 dB SPL SNR was also higher than the desired 0.50 (*M* = 0.73). It was important to ensure that baseline performance was not too high so that participants had room to improve with the addition of congruent context. The high accuracy in the present study using the SNRs from Failes et al. (2020) was unsurprising given that our SPIN task was a four-alternative forced-choice test as opposed to the open-set response format used by Failes et al. It was determined that an SNR of −10 dB SPL for younger adults and −7 dB SPL for older adults would achieve approximately equal performance across groups with accuracy that left room for improvement from the baseline condition to the congruent condition.

### Procedure

**Figure 1:**
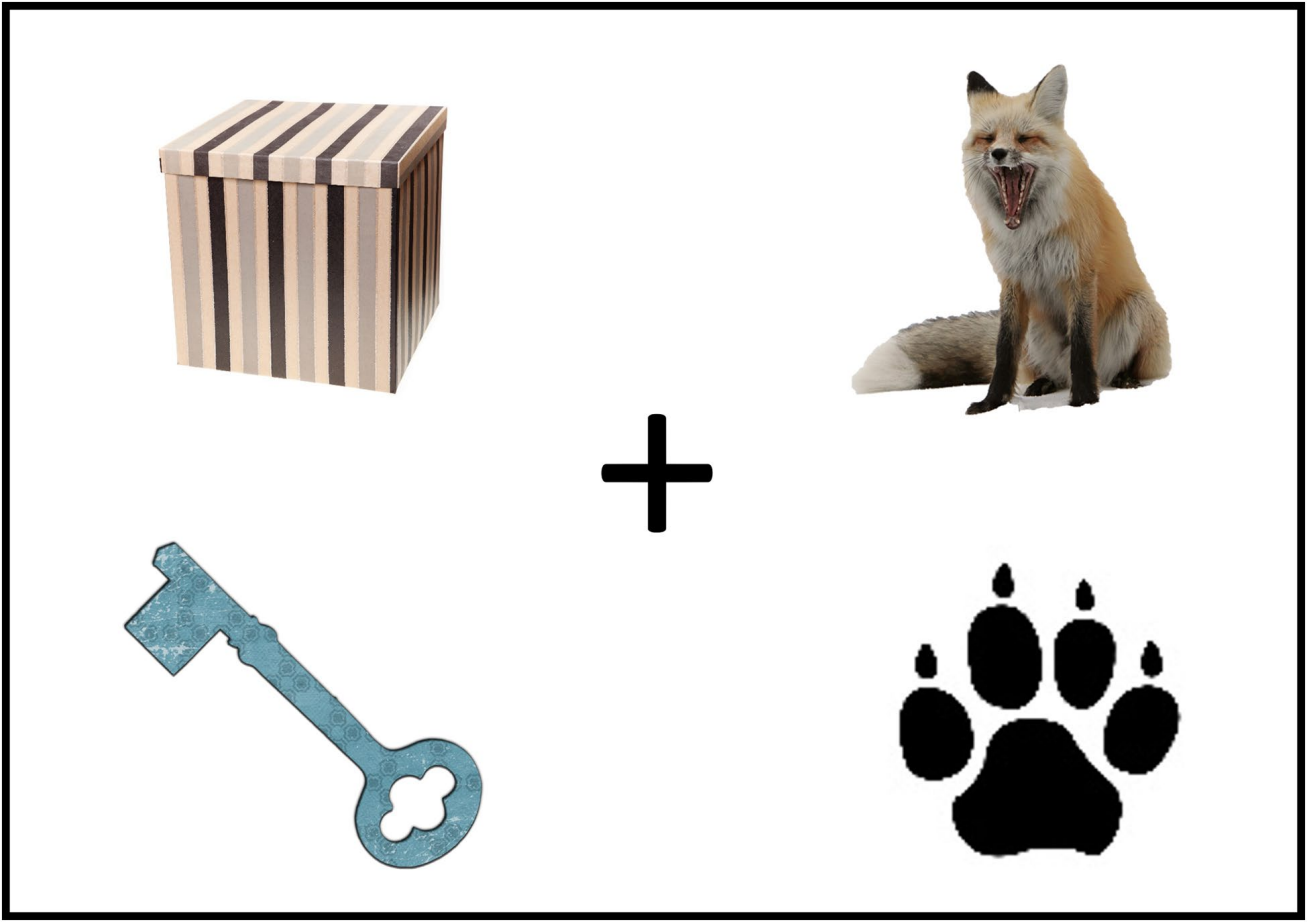
Recreation of the example screen from the visual world task for the congruent sentence She put the toys in the box. For copyright reasons the original figure cannot be reproduced, but is available upon request to the corresponding author. Images sourced from stockvault.net.

Participants first completed an audiogram inside a sound-attenuating booth. Following this, participants were seated at an EyeLink 1000 eye-tracking-enabled computer, where they completed the Shipley Vocabulary Test (Shipley, 1940) before beginning the visual world task. Participants placed their chins on a chinrest with their foreheads against a forehead rest to complete the visual world task. The distance from the back of the forehead rest to the eyepiece of the eye-tracker, which was positioned in front and below the computer monitor, was 52.07 cm and the distance from the back of the forehead rest to the center of the computer monitor was 57.78 cm. Participants first completed a study phase wherein each image to be shown in the visual world task was shown with the word the image was meant to depict for 2,000 ms to ensure that participants knew what each image represented. Participants then completed three practice trials, followed by 90 test trials, equally divided between the three sentence types (baseline/congruent/incongruent). Each trial began when the participant clicked on a central fixation cross. On each trial, a sentence was played through headphones with the final word in noise, and the four images associated with that sentence were presented on screen, one randomly assigned to each quadrant (see Figure 1). Participants were instructed to look at and click on the image corresponding to the word presented in noise. They were also told that they could move their eyes freely about the screen as long as the images were displayed but would only be able to click on an image once the sentence finished. Participants were specifically instructed that the sentence contexts could sometimes be misleading and were given examples (not included in the main study) of a sentence from each of the three conditions. Images remained on screen until the participant clicked on one of them. After clicking on an image, participants clicked on a number from one to five to indicate their confidence that they had selected the correct image, where one indicated a complete guess and five indicated absolute certainty.

The eye-tracker was calibrated immediately before test trials to ensure accurate eye-tracking. For the calibration task, dots appeared at 13 different locations on the screen and participants were tasked with fixating on each dot when it appeared and continuing to look at the dot until it disappeared. Participants completed the calibration task until it had been rated a “good” calibration by the EyeLink program, then completed an additional validation calibration to ensure that calibration was consistently accurate.

## Results

### Accuracy

We first analyzed age differences in accuracy across the baseline, congruent, and incongruent conditions with mixed-effects logistic regression using the glmer function from the *lme4* package in R (Bates et al., 2015). The dependent variable in this model was trial-by-trial accuracy. The model included an intercept term corresponding to the odds of an accurate response for younger adults in the baseline condition, two dummy coded variables indicating the change in the odds of an accurate response from the baseline condition to the congruent and incongruent conditions for younger adults, a group variable representing the change in odds of an accurate response from younger to older adults in the baseline condition, and the interaction of group with the congruent and incongruent condition dummy codes to determine whether the change in the odds of an accurate response from the baseline condition to the congruent and incongruent conditions differed between younger and older adults. The odds of an accurate response in the baseline condition and the change in odds of an accurate response from the baseline to the congruent and incongruent conditions were allowed to vary randomly across subjects, and the odds of an accurate response were allowed to vary randomly across items (i.e., target words).

**Figure 2:**
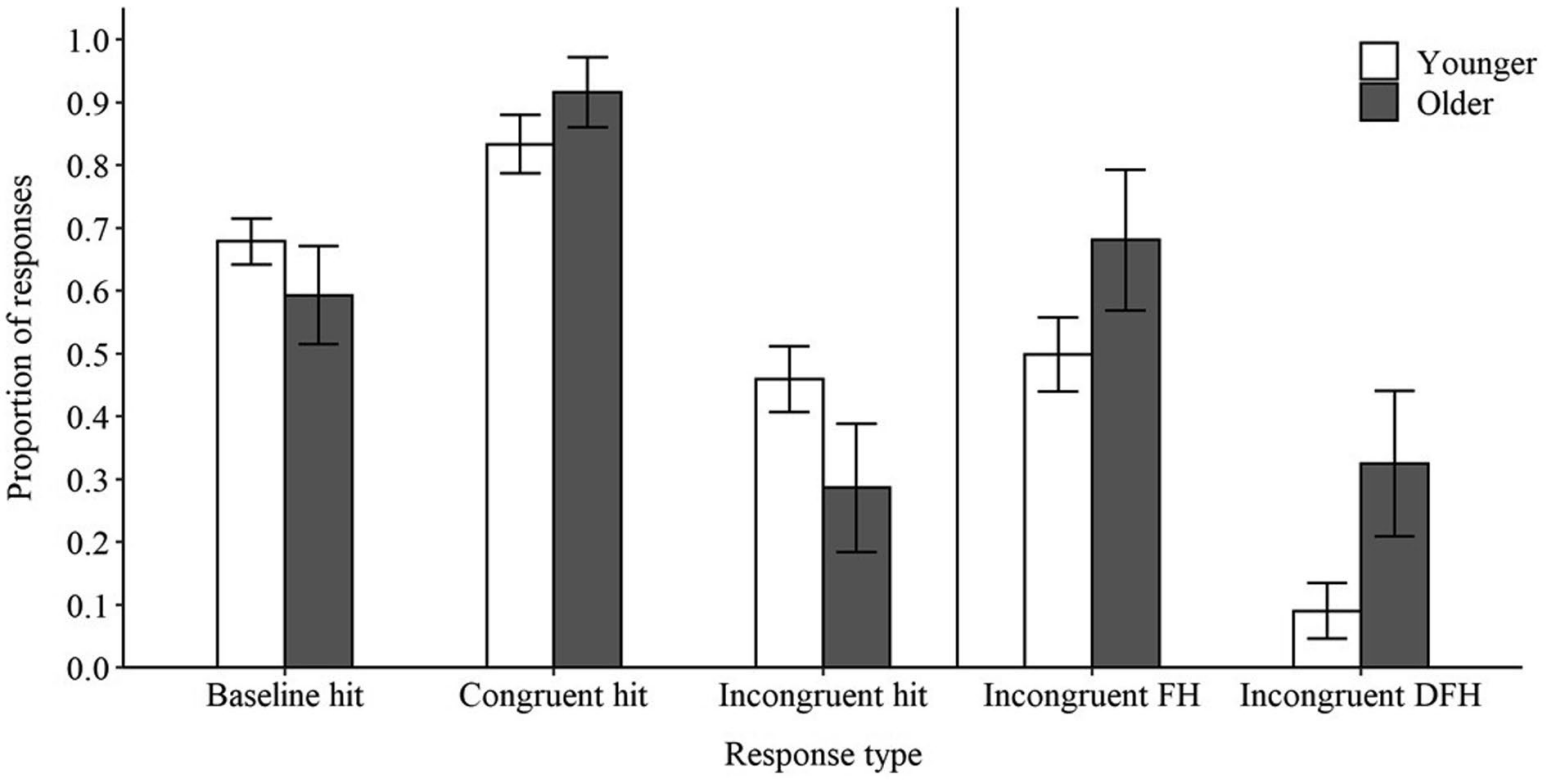
Average proportion of hits (left side) and susceptibility to false hearing (FH; right side) and dramatic false hearing (DFH) for younger and older adults. Error bars represent 95% confidence intervals.

Average accuracy in the baseline, congruent, and incongruent conditions is presented in Figure 2. Younger adults displayed better baseline accuracy than older adults [*Odds Ratio* (*OR*) = 0.64, *z* = −2.05, *p* < 0.05], indicating that the SNR manipulation did not successfully equate performance in the baseline condition across groups. As expected, younger adults displayed improved performance in the congruent condition (*OR* = 3.17, *z* = 2.67, *p* < 0.01) and poorer performance in the incongruent condition (*OR* = 0.29, *z* = −3.25, *p* < 0.01) relative to the baseline condition. However, older adults experienced significantly greater benefit in the congruent condition (*OR* = 6.62, *z* = 4.15, *p* < 0.001) and significantly greater detriment to performance in the incongruent condition (*OR* = 0.43, *z* = −2.63, *p* < 0.01) relative to baseline than did younger adults. Although it may be argued that older adults’ greater benefit in the congruent condition relative to younger adults could have resulted because younger adults had less room for improvement due to their better baseline performance, this explanation is unlikely since younger adults did not approach ceiling performance in the congruent condition (see Figure 2). These results replicate those from past studies of false hearing (Rogers et al., 2012; Sommers et al., 2015; Failes et al., 2020) and support the argument that older adults’ performance was influenced more by available contextual cues than was that of younger adults.

### False Hearing

To determine whether younger and older adults differed in susceptibility to false hearing, we created another mixed-effects logistic regression model predicting trial-by-trial false hearing on a subset of data that included only incongruent trials. The predictors in this model were an intercept term corresponding to the odds of false hearing in the younger adult group and an age group variable corresponding to the change in the odds of false hearing from the younger adult group to the older adult group. The odds of experiencing false hearing were allowed to vary randomly across subjects and across items.

As shown in Figure 2, both younger and older adults experienced false hearing. The odds that a younger adult would experience false hearing on incongruent trials was equivalent to the odds of giving any other possible response on incongruent trials (i.e., a correct response or erroneously choosing one of the foils) combined (*OR* = 0.98, *z* = −0.05, *p* > 0.05). The odds that an older adults would experience false hearing were approximately four times greater than the odds for younger adults, which was significant (*OR* = 4.04, *z* = 3.47, *p* < 0.001).

We also analyzed the odds of experiencing false hearing with maximum confidence—referred to in past studies as *dramatic false hearing* (Rogers et al., 2012; Sommers et al., 2015) —in younger and older adults (see Figure 2). The odds that a younger adult would experience dramatic false hearing was far less than the odds of giving any other possible response in the incongruent condition (*OR* = 0.03, *z* = −8.14, *p* < 0.001). Similar to previous studies (Rogers et al., 2012; Sommers et al., 2015), the odds that older adults would experience dramatic false hearing was more than 10 times greater than for younger adults (*OR* = 10.46, *z* = 4.97, *p* < 0.001). Thus, despite having an image depicting the correct target word presented on screen, both younger and older adults incorrectly reported hearing the contextually predicted word in over 50% of incongruent sentences, with older adults doing so more often and being far more likely to report maximum confidence in these errors than younger adults.

### Confidence

#### Accurate Responses

To determine whether sentence condition and age group differences existed for confidence in accurate responses, we created a linear mixed-effects regression model using the lmer function from the *lme4* package in R (Bates et al., 2015) using a subset of data that included only accurate responses. As in the accuracy analyses, the model included an intercept term corresponding to confidence in accurate responses for younger adults in the baseline condition, two dummy coded variables indicating the change in confidence from the baseline condition to the congruent and incongruent conditions for younger adults, a group variable representing the change in confidence from younger to older adults in the baseline condition, and the interaction of group with the congruent and incongruent condition dummy codes to determine whether the change in confidence from the baseline condition to the congruent and incongruent conditions differed between younger and older adults. Confidence in the baseline condition and changes in confidence from the baseline condition to the congruent and incongruent conditions were allowed to vary randomly across subjects, and confidence was allowed to vary randomly across items.

**Figure 3:**
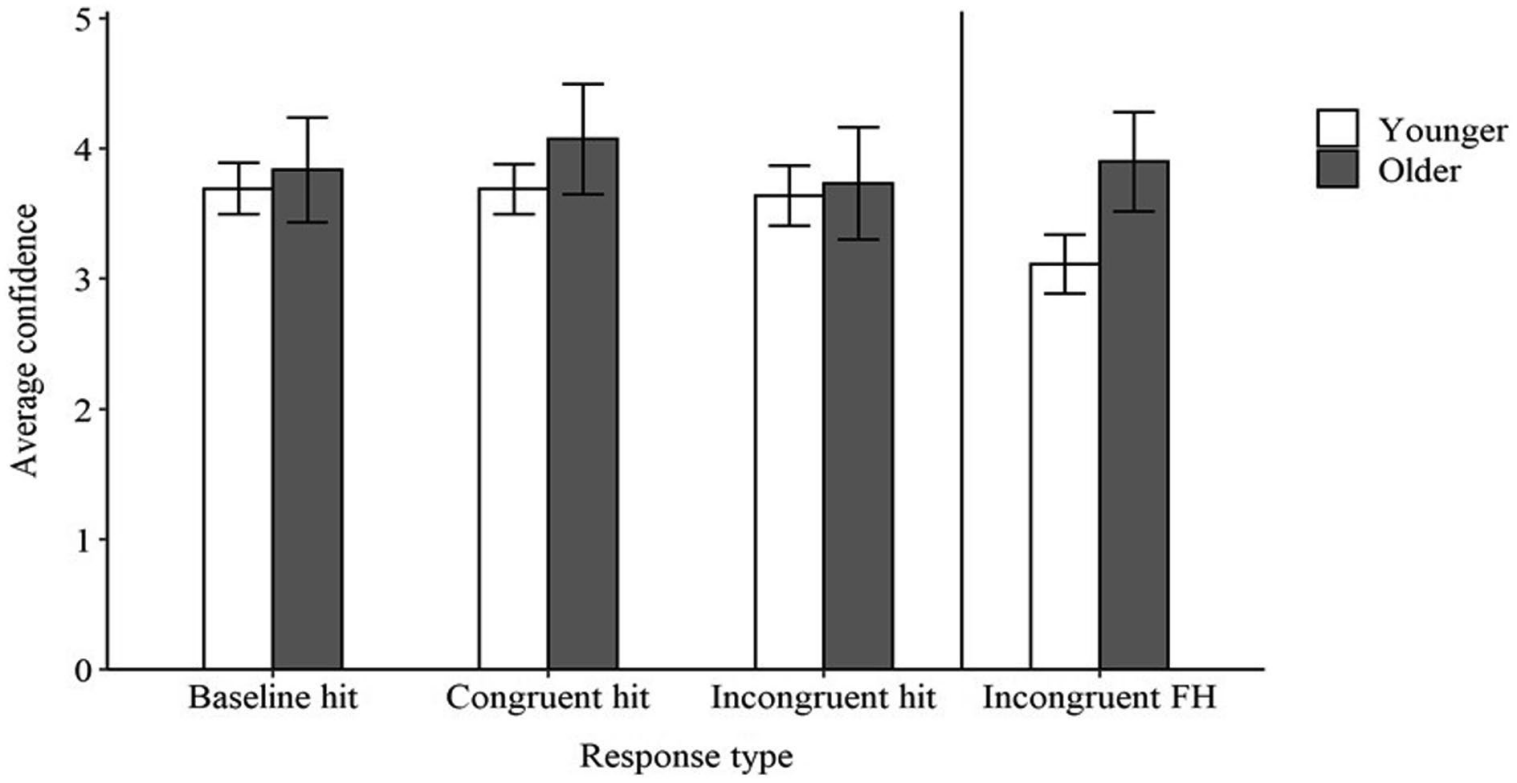
Average confidence in hits in the baseline, congruent, and incongruent conditions, and in cases of false hearing (FH) in the incongruent conditions for younger and older adults. Error bars represent 95% confidence intervals.

Average confidence in accurate responses (hits) in the baseline, congruent, and incongruent conditions is presented in Figure 3. Younger adults expressed confidence slightly above a neutral rating for accurate responses in the baseline condition, with the model estimating an average confidence of 3.53 out of 5. Younger and older adults’ confidence did not differ in the baseline condition [*Estimated Difference* (*ED*) = 0.06, *t* = 0.32, *p* > 0.05]. Younger adults’ confidence for accurate responses did not differ in either the congruent condition (*ED* = 0.10, *t* = 0.43, *p* > 0.05) or the incongruent condition (*ED* = −0.23, *t* = −1.10, *p* > 0.05) relative to the baseline condition. Additionally, the difference in confidence between the baseline and incongruent conditions did not differ in older adults relative to younger adults (*ED* = −0.02, *t* = −0.18, *p* > 0.05). However, there was a significant interaction suggesting that older adults’ confidence increased to a greater degree than that of younger adults from the baseline condition to the congruent condition (*ED* = 0.39, *t* = 2.18, *p* < 0.05). Overall, these findings suggest that participants’ confidence in accurate responses remained quite stable regardless of the context condition, aside from higher confidence in the congruent condition by older, relative to younger, adults. This differs from in past studies (Rogers et al., 2012; Sommers et al., 2015) where both younger and older adults have demonstrated lower confidence in accurate responses in the baseline condition than in the congruent condition. It is possible that changing to a four-alternative forced-choice paradigm, rather than the open-set response format used in the earlier studies, resulted in the consistently high confidence in accurate responses in the present study. For example, if a participant thought they heard the word *box*, they might be more confident in that response because an image of a box was among the four options on screen. Therefore, the availability of images on the screen may have increased confidence in accurate perceptions.

#### False Hearing Responses

We then conducted a second linear mixed-effects regression analysis to determine whether younger and older adults differed in their confidence in cases of false hearing. This model was conducted on a subset of data that included only cases of false hearing on incongruent trials. The model included an intercept term corresponding to younger adults’ confidence in cases of false hearing and a group variable indicating the change in confidence from younger to older adults. Confidence in cases of false hearing was allowed to vary randomly across subjects and items.

Although there was little difference between age groups for confidence in accurate responses, younger and older adults did differ in their confidence in cases of false hearing (see Figure 3). Younger adults expressed approximately neutral confidence in cases of false hearing, with the model estimating an average confidence of 3.12 out of 5. Older adults’ estimated average confidence in cases of false hearing was 3.93, which was significantly higher than the confidence displayed by younger adults (*t* = 4.08, *p* < 0.001). Thus, older adults were both more susceptible to and more confident in cases of false hearing than were younger adults.

### Fixation Analyses

To determine changes in the proportion of fixations on each image across time in the visual world task, linear mixed-effects regression was used to analyze the proportion of fixations on each image following the analyses used in a recent eye-tracking study that employed a similar visual world paradigm (Ito et al., 2018). Fixations on locations of the screen other than one of the four images (e.g., on the fixation cross) were not included when calculating the proportion of fixations on each image. Separate analyses were conducted for each sentence type (baseline, congruent, incongruent). Both accurate and inaccurate responses were included in analyses unless otherwise noted. Sentences were divided into three 2,000-ms bins for fixation analyses. The first bin started from 500 ms after the start of the trial since there were very few fixations on any of the images before this time (participants tended to still be looking at the central fixation cross). The second time bin started 2,500 ms into the trial and continued until just before the target word was presented. The third time bin started 4,500 ms into the trial, exactly when the target word onset. These three bins allowed us to determine the proportion of fixations on each image early in the sentence (bin 1), late in the sentence but before the target word was presented (bin 2), and from the presentation of the target word onwards (bin 3). Although other analytic approaches, such as growth-curve analysis, are often used to examine the time course of spoken word recognition in the visual world paradigm, they can sometimes obscure important changes at specific time points, such as the transition between preceding context and target word. We therefore elected to bin the eye-tracking data as described to facilitate comparisons at specific points in the sentence using the raw eye-tracking data. Each mixed-effects model had 24 dummy coded variables corresponding to the four picture types (target image, alternative image, and two foil images) within each age group (younger/older adults) at each time bin as fixed effects predicting the proportion of fixations. Proportion of fixations was allowed to vary randomly across subjects and items for each image type within each time bin. Linear combinations of the fixed effects were tested using the *multcomp* package in R (Hothorn et al., 2008) to determine how the proportion of fixations on each image changed over time, whether the proportion of fixations differed across image types within time bins, and whether these effects differed across age groups. Since the target image and the alternative image (i.e., the contextually predicted image in incongruent sentences) were of primary interest to our hypotheses, we will focus on fixation trends for these image types in the Results section. Analyses pertinent to the two foil images are presented in the Supplementary Material.

#### Baseline Sentences

**Figure 4:**
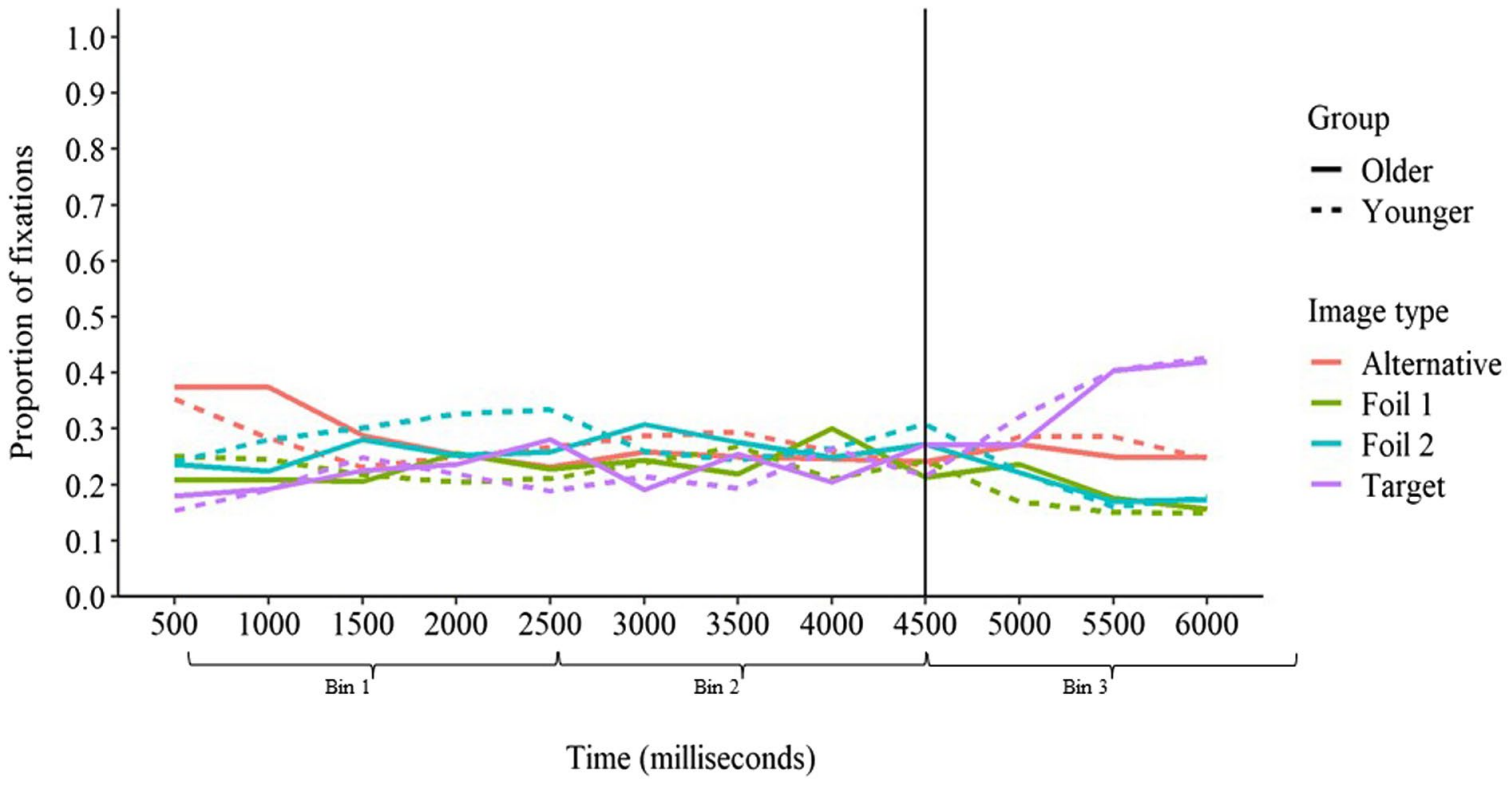
Proportion of fixation on each image type over time in baseline sentences for younger and older adults. The vertical line at 4,500 ms represents the onset of the target word.

The proportion of fixations over time for baseline sentences is presented in Figure 4. For baseline sentences, we predicted that fixations on the target image would not increase until time bin 3 since there were no contextual cues in baseline sentences to afford anticipatory activation to any particular response. This prediction was supported by the fixation analysis. In baseline sentences, there was no difference in fixations on the target image from time bin 1 to time bin 2 (*ED* = 0.03, *z* = 1.48, *p* > 0.05), but fixations on the target image increased from time bin 2 to time bin 3 (*ED* = 0.27, *z* = 12.80, *p* < 0.001). There was no interaction with age group for the difference from time bin 1 to time bin 2 (*ED* = 0.00, *z* = 0.08, *p* > 0.05) or from time bin 2 to time bin 3 (*ED* = 0.02, *z* = 0.74, *p* > 0.05). Therefore, as predicted, both younger and older adults only increased fixations on the target image after the target word had been presented in baseline sentences, demonstrating that neither group experienced anticipatory activation of the target word when no contextual cues were present.

We next compared the relative proportion of fixations on the target image and the alternative image within each time bin. Participants demonstrated a greater proportion of fixations on the alternative image than the target image in time bins 1 (*ED* = −0.18, *z* = −9.31, *p* < 0.001) and 2 (*ED* = −0.10, *z* = −4.84, *p* < 0.001), but the opposite was true in time bin 3 (*ED* = 0.20, *z* = 9.74, *p* < 0.001). There were no interactions with age group for differences in fixations between the target and alternative images (*p*s > 0.05). This suggests that although the alternative word was activated to a greater degree than the target word before the target word was presented—potentially due to minor differences in word frequency between the target word and alternative word (see Luce and Pisoni, 1998; Dahan et al., 2001; Dahan and Gaskell, 2007; Revill and Spieler, 2012) or differences in characteristics of the target image and alternative image themselves—the target word became more highly activated than the alternative word once the target word was presented.

#### Congruent Sentences

**Figure 5:**
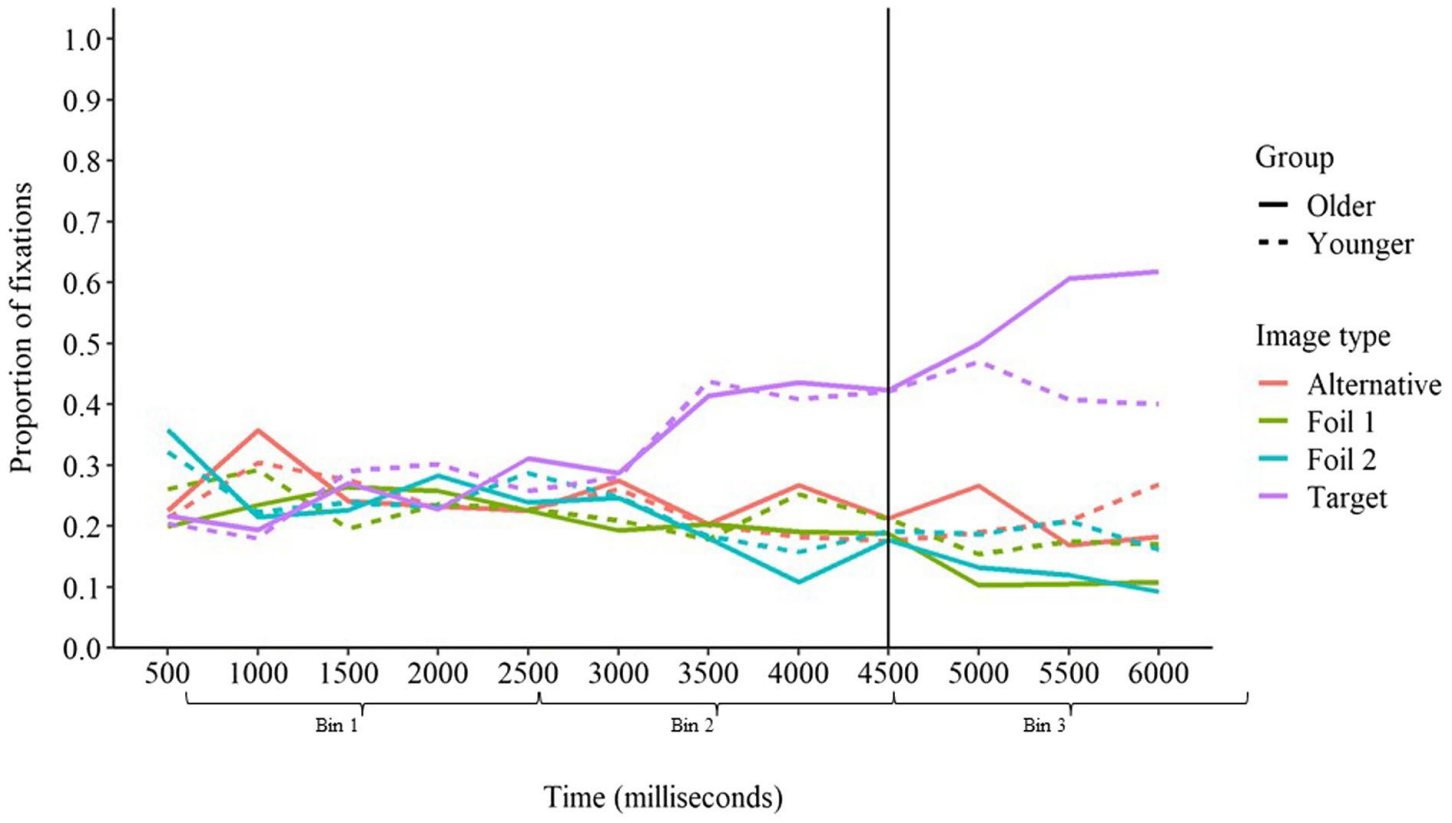
Proportion of fixations on each image type over time in congruent sentences for younger and older adults. The vertical line at 4,500 ms represents the onset of the target word.

For congruent sentences, we predicted that both age groups would begin looking toward the contextually predicted target image before the target word was presented. Additionally, we predicted that older adults might increase their fixations on the target image to a greater degree than younger adults, reflecting increased influence of context over responding. As can be seen in Figure 5, these predictions were mostly supported by the fixation data. Fixations on the target image increased from time bin 1 to time bin 2 (*ED* = 0.25, *z* = 12.98, *p* < 0.001) and again from time bin 2 to time bin 3 (*ED* = 0.26, *z* = 12.81, *p* < 0.001). Whereas there was no interaction between age group and the change in fixations from time bin 1 to time bin 2 (*ED* = −0.01, *z* = −0.27, *p* > 0.05), there was an interaction with age group for the change in fixations from time bin 2 to time bin 3 (*ED* = −0.15, *z* = −7.17, *p* < 0.001). For younger adults, there was a significant increase in proportion of fixations on the target image from time bin 2 to time bin 3 (*ED* = 0.06, *z* = 4.13, *p* < 0.001), but this increase was significantly greater for older adults (*ED* = 0.20, *z* = 13.69, *p* < 0.001). Thus, both younger and older adults increased fixations on the target image before the target word was presented, demonstrating anticipatory activation of the target word based on available contextual cues. Older adults increased fixations on the target image to a greater degree than younger adults, but only after the target word had been presented. This suggests that younger and older adults formed context-based expectations at a similar rate, but older adults became more fixated on the contextually predicted response once additional support for this response was provided by presentation of the target word. The greater increase in fixations on the target image after the target word was presented by older, relative to younger, adults suggests that older adults may use the auditory signal to confirm their context-based expectations. When the auditory signal supported the word they expected to hear, older adults become increasingly fixated on that response option, whereas younger adults may have been more cautious and considered alternative options.

As was true in baseline sentences, the alternative image received a greater proportion of fixations than the target image in time bin 1 (*ED* = −0.06, *z* = −3.20, *p* < 0.01). However, as sentence context continued to be introduced in bin 2, the target image began to receive a greater proportion of fixations than the alternative image (*ED* = 0.27, *z* = 13.66, *p* < 0.001). The target image’s advantage in terms of fixations increased further in time bin 3 (*ED* = 0.55, *z* = 26.89, *p* < 0.001). Although the difference in fixations between the target and alternative images did not interact with age group for time bin 1 or 2 (*p*s > 0.05), there was a significant interaction for time bin 3 (*ED* = −0.21, *z* = −10.02, *p* < 0.001). This interaction indicated that although younger adults devoted a greater proportion of fixations to the target image relative to the alternative image in time bin 3 (*ED* = 0.17, *z* = 12.12, *p* < 0.001), this difference was substantially greater for older adults (*ED* = 0.38, *z* = 25.71, *p* < 0.001). These findings indicate that although the alternative word was initially more highly activated than the target word, the activation of the target word exceeded that of the alternative word as support for that word was introduced *via* semantic context and the phonological characteristics of the target word.

#### Incongruent Sentences

Finally, for incongruent sentences, we had again predicted that both younger and older adults would look toward the contextually predicted image before the target word was presented. In incongruent sentences, the alternative word was predicted by context as opposed to the target word, so we predicted that fixations on the alternative image would increase before the target word was presented. Here again we predicted that older adults would increase fixations on the alternative image to a greater degree than younger adults, reflecting greater influence of context over responding. After the target word was presented, we predicted that fixations on the alternative image would decrease and fixations on the target image would increase for younger adults as they realized that the presented target word differed from the contextually predicted word. However, we predicted that older adults would be less likely than younger adults to shift their focus toward the target image, instead either maintaining or increasing fixations on the alternative image, reflecting older adults’ poorer ability to suppress the expected response and increased susceptibility to false hearing in incongruent sentences.

**Figure 6:**
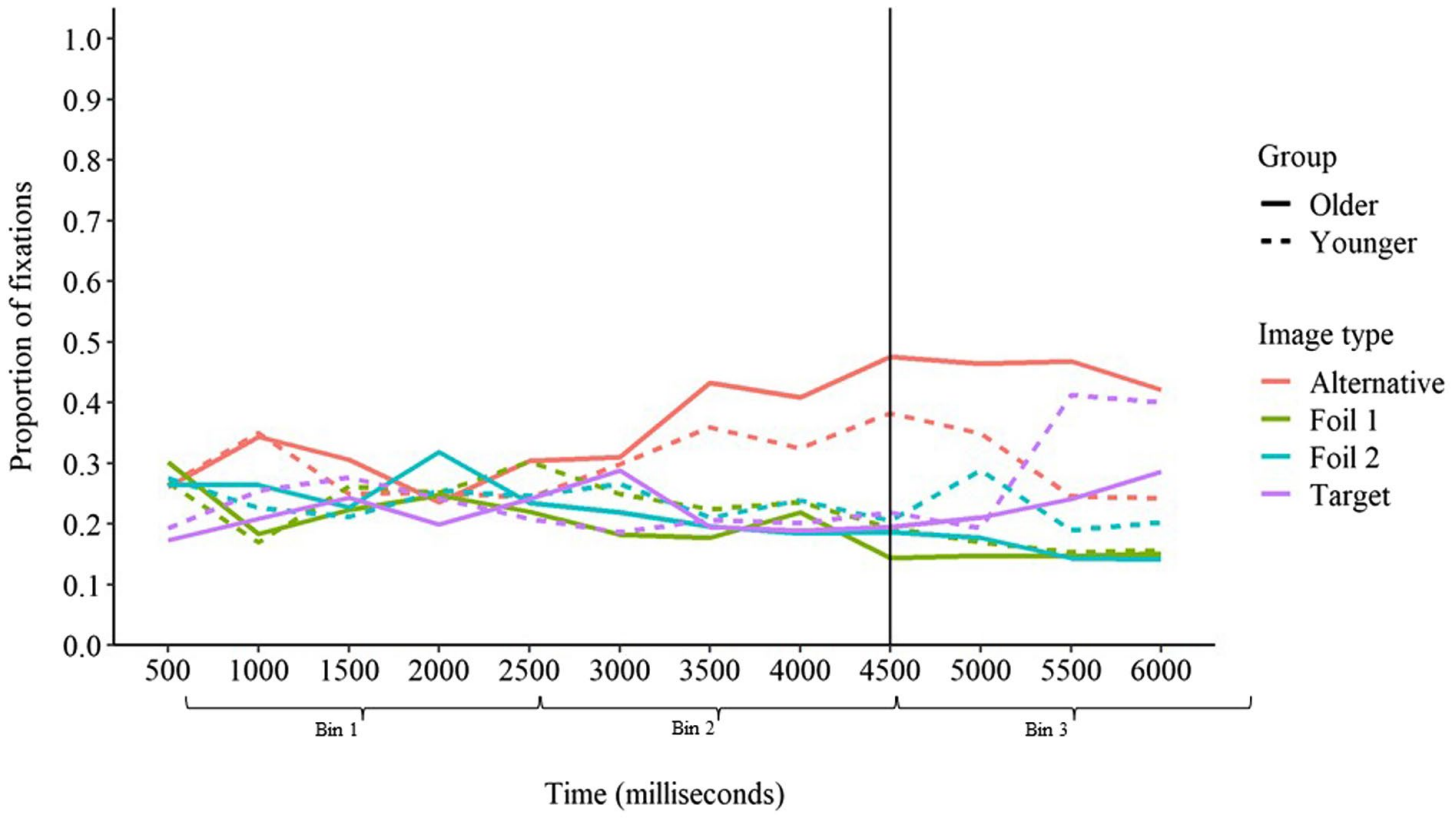
Proportion of fixations on each image type over time in incongruent sentences for younger and older adults. The vertical line at 4,500 ms represents the onset of the target word.

Average fixations over time in incongruent sentences can be seen in Figure 6. As predicted, the proportion of fixations on the alternative image increased from time bin 1 to time bin 2 (*ED* = 0.17, *z* = 8.87, *p* < 0.001), whereas fixations on the target image did not increase (*ED* = −0.02, *z* = −1.07, *p* > 0.05). There was no interaction with age group for the difference between time bin 1 and time bin 2 for fixations on the alternative image (*ED* = −0.01, *z* = −0.70, *p* > 0.05), but there was a marginally significant interaction for fixations on the target image (*ED* = −0.04, *z* = −1.92, *p* = 0.05). This interaction reflected that younger adults significantly decreased fixations on the target image from time bin 1 to time bin 2 (*ED* = −0.03, *z* = −2.24, *p* < 0.05) whereas older adults’ fixations on the target image did not change (*ED* = 0.01, *z* = 0.57, *p* > 0.05). Interestingly, fixations increased from time bin 2 to time bin 3 for both the alternative image (*ED* = 0.04, *z* = 2.03, *p* < 0.05) and the target image (*ED* = 0.16, *z* = 7.65, *p* < 0.001). However, as predicted, there were significant interactions with age group for the difference in fixations from time bin 2 to time bin 3 for both the alternative image (*ED* = −0.14, *z* = −6.90, *p* < 0.001) and the target image (*ED* = 0.12, *z* = 5.88, *p* < 0.001). Younger adults decreased fixations on the alternative image (*ED* = −0.05, *z* = −3.51, *p* < 0.001) and increased fixations on the target image (*ED* = 0.14, *z* = 9.75, *p* < 0.001) from time bin 2 to time bin 3. Older adults, however, increased fixations on the alternative image (*ED* = 0.09, *z* = 6.20, *p* < 0.001) and did not increase fixations on the target image (*ED* = 0.02, *z* = 1.23, *p* > 0.05) from time bin 2 to time bin 3. This supports the conclusion that older adults were less able to suppress the activation of the predicted response in the incongruent condition than were younger adults.

As was true for both baseline and congruent sentences, the alternative image initially received a greater proportion of fixations than the target image (*ED* = −0.12, *z* = −6.27, *p* < 0.001). This difference increased in time bin 2 as contextual cues supporting the alternative image were introduced (*ED* = −0.31, *z* = −15.74, *p* < 0.001). There was no interaction with age group for the difference between the alternative and target images in time bin 1 or 2 (*p*s > 0.05). The alternative image continued to receive a greater proportion of fixations, on average, relative to the target image in time bin 3 (*ED* = −0.20, *z* = −9.56, *p* < 0.001), but this was qualified by a significant interaction with age group (*ED* = 0.26, *z* = 12.44, *p* < 0.001). Although the alternative image did indeed receive a greater proportion of fixations than the target image in time bin 3 for older adults (*ED* = −0.23, *z* = −15.61, *p* < 0.001), the opposite was true for younger adults, with the target image receiving a greater proportion of fixations (*ED* = 0.03, *z* = 2.02, *p* < 0.05). This suggests that younger adults were better able to reduce the activation of the contextually predicted (but incorrect) word below that of the unpredicted target word than were older adults on incongruent trials, which may help to explain why older adults were more susceptible to false hearing.

**Figure 7:**
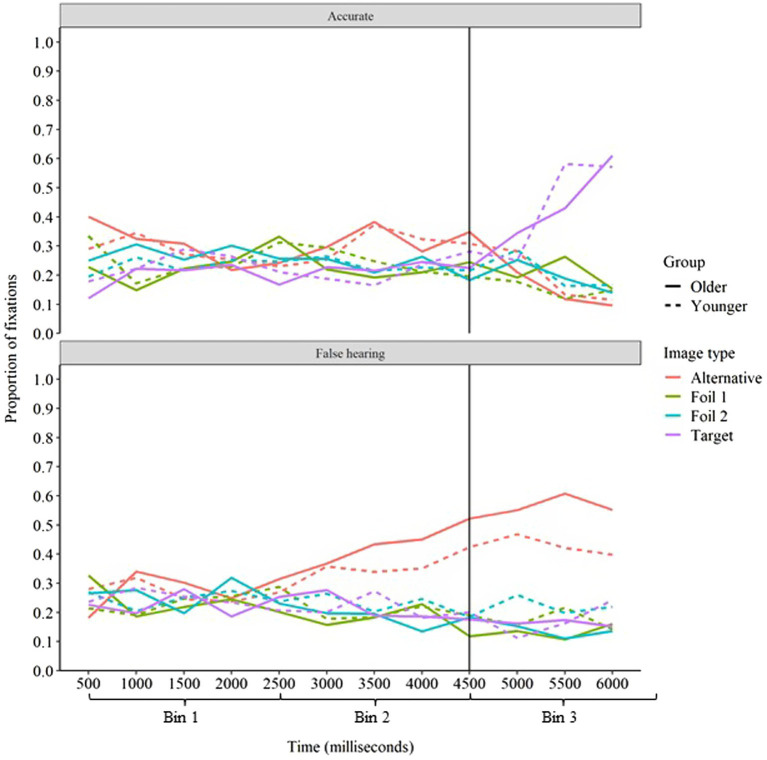
Proportion of fixations on each image type over time in incongruent sentences for younger and older adults on accurate and false hearing. The vertical line at 4,500 ms represents the onset of the target word.

To better understand how anticipatory activation of the contextually predicted word contributed to the likelihood of false hearing, we next compared changes in fixations on the target and alternative images over time for accurate responses and false hearing responses on incongruent trials. Average fixations on each image type for younger and older adults for accurate and false hearing responses are displayed in Figure 7.

The change in fixations on the alternative image from bin 1 to bin 2 differed significantly across accurate and false hearing trials (*ED* = −0.19, *z* = −3.17, *p* < 0.01). Fixations on the alternative image did not change from bin 1 to bin 2 for accurate trials (*ED* = −0.01, *z* = −0.28, *p* > 0.05), whereas fixations on the alternative image increased substantially from bin 1 to bin 2 for false hearing trials (*ED* = 0.18, *z* = 4.27, *p* < 0.001). This finding is interesting, as it suggests that there was relatively little anticipatory activation of the contextually predicted word on incongruent trials in which participants responded accurately relative to those in which false hearing occurred. The proportion of fixations on the target image did not change from bin 1 to bin 2 (*ED* = −0.06, *z* = −0.91, *p* > 0.05) and there was no difference in the change in fixations on the target image from bin 1 to bin 2 across accurate and false hearing trials (*ED* = 0.01, *z* = 0.22, *p* > 0.05).

Unsurprisingly, the change in fixations on the alternative image from bin 2 to bin 3 also differed significantly across accurate and false hearing trials (*ED* = −0.46, *z* = −7.50, *p* < 0.001). Fixations on the alternative image decreased from bin 2 to bin 3 for accurate trials (*ED* = −0.20, *z* = −4.48, *p* < 0.001), whereas fixations on the alternative image increased from bin 2 to bin 3 for false hearing trials (*ED* = 0.26, *z* = 6.16, *p* < 0.001). The change in fixations on the target image from bin 2 to bin 3 also differed significantly across accurate and false hearing trials (*ED* = 0.51, *z* = 8.38, *p* < 0.001). Fixations on the target image increased greatly from bin 2 to bin 3 for accurate trials (*ED* = 0.42, *z* = 9.56, *p* < 0.001), but decreased from bin 2 to bin 3 for false hearing trials (*ED* = −0.09, *z* = −2.19, *p* < 0.05). All interactions with age group were non-significant when considering accurate and false hearing trials separately (*p*s > 0.05).

It is interesting to note that fixations on the target image did not reach 100% on accurate trials, nor did fixations on the alternative image reach 100% on trials in which false hearing occurred. This may have resulted for several reasons. It is possible that this reflects uncertainty as to the identity of the target word, with participants continuing to consider other response options in time bin 3. It is also possible that participants might approach 100% fixations on the target image if we used shorter time bins. We analyzed our data within 2,500 ms time bins to increase statistical power while still being able to address our research questions, but the extended duration of these bins might capture fixations on other response options leading up to settling on a single image. However, even in Figure 7, which presents the fixation data in 500 ms bins, neither age group approaches 100% fixations on the target image. Thus, it is possible that fixations on the image corresponding to the participants’ response could have reached 100%, but the short duration of the time bins required to achieve this would have greatly increased the complexity of our models without improving our ability to address our research questions.

For the difference in proportion of fixations between the target image and the alternative image, there were marginally significant interactions indicating differences between accurate and false hearing trials in bins 1 (*ED* = −0.10, *z* = −1.70, *p* = 0.09) and 2 (*ED* = 0.10, *z* = 1.68, *p* = 0.09), and a significant interaction in bin 3 (*ED* = 1.08, *z* = 17.76, *p* < 0.001). Beginning with accurate trials, the alternative image received a greater proportion of fixations in both time bins 1 (*ED* = −0.17, *z* = −3.85, *p* < 0.001) and 2 (*ED* = −0.18, *z* = −4.00, *p* < 0.001), but the opposite was true in time bin 3 (*ED* = 0.44, *z* = 10.11, *p* < 0.001). On incongruent trials in which false hearing occurred, the alternative image did not differ from the target image in fixations in time bin 1 (*ED* = −0.06, *z* = −1.53, *p* > 0.05). The alternative image did, however, receive more fixations than the target image in time bin 2 (*ED* = −0.28, *z* = −6.58, *p* < 0.001), and as indicated by the significant interaction mentioned above, this difference was greater than that in bin 2 of accurate trials. Unlike for accurate trials, the alternative image also received a greater proportion of fixations than the target image in time bin 3 (*ED* = −0.64, *z* = −15.08, *p* < 0.001). Additionally, although there were no interactions with age group for any of the effects mentioned above (*p*s > 0.05), the difference in proportion of fixations between the alternative image and the target image differed in magnitude across age groups for bin 3 of trials in which false hearing occurred (*ED* = 0.15, *z* = 3.50, *p* < 0.001). Specifically, the alternative image received a greater proportion of fixations relative to the target image for younger adults (*ED* = −0.24, *z* = −8.35, *p* < 0.001), but this difference was greater for older adults (*ED* = −0.39, *z* = −12.89, *p* < 0.001). These findings are particularly important, as they suggest that participants were able to reduce the activation of the contextually predicted (but incorrect) word below that of the unpredicted target word on accurate trials but were unable to do so for trials in which false hearing occurred.

## Discussion

The current study was designed to provide a more direct test of the inhibitory deficit hypothesis of false hearing using eye-tracking as an online measure of lexical activation. Overall, the findings suggest that false hearing occurs when participants fail to reduce activation on semantically congruent, but incorrect, lexical items that are phonologically similar to target words. Using eye-tracking, we found that fixations, an index of lexical activation, increased on contextually congruent words for both younger and older adults prior to actual target word onset. When semantically congruent items were presented as targets (congruent condition), fixations to the target increased following target word onset and did so to a greater extent for older than for younger adults. Activation of the phonologically similar but semantically incongruent foil in this condition remained low for both older and younger adults. However, when a phonologically similar alternative was the target (incongruent condition), younger adults and older adults again both increased fixations to the semantically congruent (but now incorrect) item prior to target word onset. Once the target word was presented, however, younger adults were more likely than older listeners to switch fixations to the correct, but semantically incongruent target item, whereas older adults maintained high levels of fixation on the incorrect alternative. Furthermore, we found that early activation of the contextually predicted word in incongruent sentences was suppressed below the activation of the target word in cases where participants accurately reported the target word, whereas activation of the contextually predicted, but incorrect, word remained higher than the target word in cases of false hearing. Taken together, the findings suggest that older adults’ increased susceptibility to false hearing stems in part from a reduced ability to inhibit the anticipated, but incorrect, response on incongruent trials (Rogers et al., 2012; Sommers et al., 2015; Failes et al., 2020).

To our knowledge, the current study is the first investigation of false hearing with sentence materials to use a closed-set response format (where the target item was presented as one of four alternatives) rather than open-set responding. This change from open- to closed-set responding resulted in a substantially easier task for both older and younger adults as indicated by the necessity of using more difficult SNRs than those used by Failes et al. (2020) to obtain similar baseline accuracy. Nevertheless, the findings largely replicate prior studies of age-related differences in false hearing. Relative to baseline (non-predictive) sentences, older adults were more accurate than younger adults in congruent conditions—a finding that is extremely rare in studies comparing speech in noise perception in younger and older adults—but significantly less accurate than younger adults in the incongruent condition. Taken together, the results demonstrate the robust effects of context on speech perception; even when the right answer was presented to participants in an image, younger adults still chose the contextually predicted (but incorrect) option on approximately 50% of incongruent trials, whereas older adults committed these context-based errors on approximately 70% of incongruent trials.

The findings from the current study provide more direct evidence than in previous studies (Sommers et al., 2015; Failes et al., 2020) that false hearing occurs when listeners are unable to sufficiently inhibit the activation of a contextually predicted, but incorrect, target word. Increased fixations on the contextually predicted image prior to target onset in both congruent and incongruent sentences suggests that the contextually predicted word became activated even before the target word was presented. For congruent sentences, this early target activation led to high accuracy rates for both age groups, with marginally better accuracy for older than for younger adults. Although there is some evidence that older adults benefit more than younger adults from supportive semantic contexts (see Pichora-Fuller, 2008 for review) it is atypical to find better performance for older than for younger adults under conditions of equal audibility. In the present study, this finding in the congruent condition indicates greater reliance on context-based responding for older than for younger adults, a proposal that is further supported by the eye-tracking results showing a significantly greater proportion of fixations to the target image in bin 3 in the congruent condition for older than younger adults.

Support for the role of inhibitory control as a factor contributing to false hearing comes from both accurate and inaccurate responding on the incongruent trials. When participants were correct on incongruent trials, both younger and older adults were able to reduce the activation of the contextually predicted word below that of the target word. In contrast, when participants selected the (inaccurate) alternative on incongruent trials (i.e., exhibited false hearing), fixations on that image did not fall below those for the actual target word, suggesting that participants failed to inhibit activation on the alternative item. Therefore, the eye-tracking data in the present study provide the first direct evidence for the theory that false hearing results when individuals are unable to reduce the activation of a contextually predicted (but incorrect) response below the activation of the correct response.

The eye-tracking data in the present study may also shed light on what aspects of inhibitory control contribute to false hearing. Previous studies have suggested that there are three distinct functions of inhibitory control: controlling what information enters working memory, removing information that is no longer relevant from working memory, and suppressing prepotent, but incorrect, responses (Kramer et al., 1994; Zacks and Hasher, 1997; Lustig et al., 2007). We will refer to these as the information gating, information removal, and response suppression aspects of inhibitory control, respectively. When looking at the eye-tracking data collapsed across all incongruent trials (see Figure 6), our data suggest that age differences in susceptibility to false hearing may be influenced primarily by either the response suppression or information removal aspects of inhibitory control. If the information gating aspect of inhibitory control contributed to age differences in susceptibility to false hearing, then we would expect younger and older adults to differ in the degree to which the contextually predicted response became activated before the target word was presented, indexing differences in the ability to control contextual information entering working memory. Instead, we found that the proportion of fixations to the contextually predicted image in the incongruent condition differed between younger and older adults only after the target word was presented. This suggests that contextual information was allowed to enter working memory to a similar degree across age groups, but older adults were either less able to remove this task-irrelevant information or were less able to suppress the contextually predicted response than were younger adults.

Recently, Van Os et al. (2021) have argued against the contribution of cognitive declines, particularly inhibitory abilities, as contributing to either age or individual differences in false hearing. Specifically, using open-set responding and sentence materials similar to those of Sommers et al. (2015), Van Os et al. reported that both older and younger adults misperceived sentence-final items as consistent with a preceding context. In contrast to prior studies of false hearing (Sommers et al., 2015; Failes et al., 2020), however, they did not report high confidence in those misperceptions. Moreover, Van Os et al. reported that the frequency of such misperceptions varied as a function of how well participants could access acoustic information; conditions that increased access to the information in speech signals, such as more favorable SNRs and easier phonetic discriminations, led to fewer misperceptions than those where listeners had less access to such information. Consequently, they argued that responding (incorrectly) on the basis of context should be considered a form of rational language comprehension in which the relative weighting of top-down (context) and bottom-up (acoustic) information varies rationally. When access to acoustic information is limited, listeners place greater emphasis on context. Conversely, when acoustic information is readily available (e.g., listening in quiet), listeners appropriately place greater emphasis on acoustic cues as a basis for responding.

In contrast to claims made by Van Os et al. (2021), however, finding that mishearing (the term used by Van Os et al., 2021) or false hearing (the term used in prior studies; Rogers et al., 2012; Sommers et al., 2015; Failes et al., 2020) varies inversely with access to acoustic cues is entirely consistent with an inhibition-based account of false hearing. Recall that activation-competition models of spoken word recognition (Luce and Pisoni, 1998; Gaskell and Marslen-Wilson, 2002) propose that activation of a particular lexical item is directly proportional to the match between the incoming speech signal and stored lexical representations. When access to the acoustic signal is readily available (e.g., favorable SNRs, normal hearing thresholds), the target item will receive considerably higher activation than other lexical competitors and demands on inhibition will be minimal. In contrast, under more difficult listening conditions, activation of targets and competitors will be more similar, and correct identification will require increased inhibition of competitors. Thus, both rational comprehension and inhibitory deficit accounts would predict increased false hearing (or misperceptions) with less favorable SNRs and more difficult phonetic discriminations –exactly the pattern reported by Van Os et al. (2021) and by Rogers et al. (2012) in their study of false hearing as a function of SNR.

In fact, we see the findings from Van Os et al. (2021) as complementary to the current results. The rational comprehension account suggests that age differences in misperceptions or false hearing result because older adults, rationally, rely on context to a greater extent than younger adults. The eye-tracking results in the current study are consistent with this proposal; despite explicit warnings that context could be misleading and a greater percentage of trials where it was misleading, older adults fixated on the semantically consistent item more than younger adults in both the congruent and incongruent conditions. Our proposal regarding the role of inhibition simply extends this account by proposing that younger adults are better able than older adults to inhibit this activation on incorrect, but semantically congruent, items than are older adults, resulting in a greater frequency of false hearing.

One limitation of the current study is that it did not include direct measures of inhibition or other cognitive abilities that may be important contributors to individual differences in the costs and benefits of semantic context. Huettig and Janse (2016), for example, found that individual differences in both working memory and processing speed were predictive of anticipatory eye movements in a visual world paradigm examining the benefits of gender marking of articles that preceded a target word in Dutch. An important direction for future studies examining the contributions of different types of supportive contexts, including semantic information and gender marking, is to include a battery of cognitive measures that can be used to establish factors that contribute to both age and individual differences in the ability to benefit from such information.

## Conclusion

The present study shed light on the processing that underlies false hearing and differences in this processing across younger and older adults. Specifically, we presented evidence that false hearing occurs when participants fail to inhibit an incorrect response that is highly activated due to its congruence with available contextual cues. Furthermore, our results advance our understanding of sentence processing, generally. We have shown that both younger and older adults form expectations regarding what will be said in the future based on preceding semantic context, expectations which are used to fill in the blanks in speech perception caused by difficult listening conditions (e.g., background noise, hearing loss). Although contextual cues in natural speech are rarely misleading—making responding based on context an efficient speech perception strategy—our demonstration of robust rates of false hearing in both younger and older adults highlight the importance of carefully attending to how information is presented. When important information is framed by valid contextual cues, both younger and older adults may more easily hear the important information. If preceding contextual cues are misleading, however, both younger and older adults –although especially older adults– may be led astray.

## Data Availability Statement

The raw data supporting the conclusions of this article will be made available by the authors, without undue reservation.

## Ethics Statement

The studies involving human participants were reviewed and approved by Washington University School of Medicine. The patients/participants provided their written informed consent to participate in this study.

## Author Contributions

All authors listed have made a substantial, direct, and intellectual contribution to the work and approved it for publication.

## Conflict of Interest

The authors declare that the research was conducted in the absence of any commercial or financial relationships that could be construed as a potential conflict of interest.

## Publisher’s Note

All claims expressed in this article are solely those of the authors and do not necessarily represent those of their affiliated organizations, or those of the publisher, the editors and the reviewers. Any product that may be evaluated in this article, or claim that may be made by its manufacturer, is not guaranteed or endorsed by the publisher.
