# Supplementary material for: Using Eye-Tracking to Investigate an Activation-Based Account of False Hearing in Younger and Older Adults
Source: Front Psychol. 2022 May 16;13:821044. doi: 10.3389/fpsyg.2022.821044 (PMC9150819; doi:10.3389/fpsyg.2022.821044)
Supplement: Supplementary file 1 [file Data_Sheet_1.docx]

**Appendix**

Here, we present analyses of the proportion of fixations on the two foil images – which we will refer to as foil 1 and foil 2 – over the course of baseline, congruent, and incongruent sentences.

**Baseline**

Fixations did not change from time bin 1 to time bin 2 for either foil 1 (*ED* = .00, *z* = .18, *p* > .05) or foil 2 (*ED* = .01, *z* = .60, *p* > .05). From time bin 2 to time bin 3, fixations decreased significantly for both foil 1 (*ED* = -.12, *z* = -5.69, *p* < .001) and foil 2 (*ED* = -.11, *z* = -5.35, *p* < .001) as fixations on the target image increased.

Within time bin 1, participants had fewer fixations on the target image than both foil 1 (*ED* = -.05, *z* = -2.43, *p* < .05) and foil 2 (*ED* = -.06, *z* = -3.14, *p* < .01). Conversely, participants had more fixations on the alternative image than either foil 1 (*ED* = .13, *z* = 6.88, *p* < .001) or foil 2 (*ED* = .12, *z* = 6.17, *p* < .001). However, there was a significant interaction with age group for the difference between the alternative image and foil 1 (*ED* = -.04, *z* = -2.28, *p* < .05), which indicated that younger adults had more fixations on the alternative image than foil 1 (*ED* = .04, *z* = 3.44, *p* < .001), but this difference was greater for older adults (*ED* = .09, *z* = 6.15, *p* < .001). There was also a significant group interaction for the comparison of the alternative image and foil 2 (*ED* = -.05, *z* = -2.58, *p* < .05), which indicated that younger adults had more fixations on the alternative image than foil 2 (*ED* = .03, *z* = 2.69, *p* < .01), but this difference was again greater for older adults (*ED* = .08, *z* = 5.88, *p* < .001). Fixations on the two foil images did not differ during time bin 1 (*ED* = -.01, *z* = -.71, *p* > .05).

During time bin 2, participants had marginally fewer fixations on the target image than on foil 2 (*ED* = -.04, *z* = -1.99, *p* = .05), but there was no difference between the target image and foil 1 (*ED* = -.02, *z* = -.95, *p* > .05). Participants again had more fixations on the alternative image than either foil 1 (*ED* = .08, *z* = 3.88, *p* < .001) or foil 2 (*ED* = .06, *z* = 2.85, *p* < .01). There was a marginally significant interaction with age group for the difference between the alternative image and foil 1 (*ED* = .04, *z* = 1.87, *p* = .06), indicating that younger adults had more fixations on the alternative image than foil 1 (*ED* = .06, *z* = 4.36, *p* < .001), whereas these image types did not differ significantly for older adults (*ED* = .02, *z* = 1.34, *p* > .05). Fixations on the two foil images did not differ during time bin 2 (*ED* = -.02, *z* = -1.03, *p* > .05).

Finally, in time bin 3, participants had more fixations on the target image than either foil 1 (*ED* = .37, *z* = 17.59, *p* < .001) or foil 2 (*ED* = .34, *z* = 16.20, *p* < .001). Participants also had more fixations on the alternative image than either foil 1 (*ED* = .16, *z* = 7.85, *p* < .001) or foil 2 (*ED* = .14, *z* = 6.46, *p* < .001), which would be expected due to the greater phonological overlap between the alternative word and the target word. Fixations on the two foil images did not differ during time bin 3 (*ED* = -.03, *z* = -1.39, *p* > .05).

**Congruent**

Fixations decreased from time bin 1 to time bin 2 for foil 1 (*ED* = -.05, *z* = -2.39, *p* < .05) and foil 2 (*ED* = -.13, *z* = -6.46, *p* < .001). There was a marginally significant interaction for foil 2 (*ED* = .04, *z* = 1.90, *p* = .06). Younger adults decreased fixations on foil 2 from time bin 1 to time bin 2 (*ED* = -.04, *z* = -3.41, *p* < .001) but older adults decreased their fixations on this image to a greater degree (*ED* = -.08, *z* = -5.61, *p* < .001). Fixations decreased further from time bin 2 to time bin 3 for foil 1 (*ED* = -.13, *z* = -6.41, *p* < .001) and foil 2 (*ED* = -.11, *z* = -5.44, *p* < .001). There were significant interactions with age group for the change in fixations between time bin 2 and time bin 3 for foil 1 (*ED* = .06, *z* = 3.04, *p* < .01). For foil 1, younger adults reduced their fixations from time bin 2 to time bin 3 (*ED* = -.03, *z* = -2.47, *p* < .05) but older adults reduced their fixations to an even greater degree (*ED* = -.10, *z* = -6.48, *p* < .001).

Within time bin 1, participants had fewer fixations on the target image than foil 2 (*ED* = -.08, *z* = -4.03, *p* < .001) but there was no difference between the target image and foil 1 (*ED* = -.01, *z* = -.43, *p* > .05). Participants had more fixations on the alternative image than foil 1 (*ED* = .05, *z* = 2.77, *p* < .01) but there was no difference between the alternative image and foil 2 (*ED* = -.02, *z* = -.83, *p* > .05). Participants had more fixations on foil 2 than foil 1 during time bin 1 (*ED* = -.07, *z* = -3.60, *p* < .001).

During time bin 2, participants had more fixations on the target image than on either foil 1 (*ED* = .29, *z* = 14.61, *p* < .001) or foil 2 (*ED* = .30, *z* = 15.14, *p* < .001). There was a marginally significant interaction with age group for the difference between the target image and foil 2 (*ED* = -.04, *z* = -1.83, *p* = .07), indicating that younger adults had significantly more fixations on the target image than foil 2 (*ED* = .13, *z* = 9.94, *p* < .001), but this difference was greater for older adults (*ED* = .17, *z* = 11.43, *p* < .001). Fixations on the alternative image did not differ from either foil 1 (*ED* = .02, *z* = .94, *p* > .05) or foil 2 (*ED* = .03, *z* = 1.48, *p* > .05) during time bin 2. However, there was a marginally significant interaction with age group for the difference between the alternative image and foil 2 (*ED* = -.04, *z* = -1.88, *p* = .06), indicating that younger adults did not differ in their fixations on the alternative image and foil 2 (*ED* = -.00, *z* = -.30, *p* > .05), whereas older adults had more fixations on the alternative image than foil 2 (*ED* = .03, *z* = 2.26, *p* < .05). Fixations on the two foil images did not differ during time bin 2 (*ED* = .01, *z* = .54, *p* > .05), but there was a marginally significant interaction (*ED* = -.04, *z* = -1.79, *p* = .07). However, the proportion of fixations on the two foil images during time bin 2 did not differ significantly for either younger (*ED* = -.01, *z* = -.93, *p* > .05) or older adults (*ED* = .02, *z* = 1.56, *p* > .05).

Finally, in time bin 3, participants had more fixations on the target image than either foil 1 (*ED* = .68, *z* = 33.20, *p* < .001) or foil 2 (*ED* = .67, *z* = 32.76, *p* < .001). There was a significant interaction with age group for the difference between the target image and foil 1 (*ED* = -.21, *z* = -10.12, *p* < .001), indicating that younger adults had more fixations on the target image than foil 1 (*ED* = .24, *z* = 16.57, *p* < .001), but this difference was far greater for older adults (*ED* = .44, *z* = 30.17, *p* < .001). There was also a significant interaction with age group for the difference between the target image and foil 2 (*ED* = -.20, *z* = -9.95, *p* < .001), again indicating that younger adults had more fixations on the target image than foil 2 (*ED* = .23, *z* = 16.38, *p* < .001), but this difference was far greater for older adults (*ED* = .44, *z* = 29.75, *p* < .001). Participants also had more fixations on the alternative image than either foil 1 (*ED* = .13, *z* = 6.30, *p* < .001) or foil 2 (*ED* = .12, *z* = 5.87, *p* < .001), which would be expected due to the greater phonological overlap between the alternative word and the target word. Fixations on the two foil images did not differ during time bin 3 (*ED* = -.01, *z* = -.44, *p* > .05).

**Incongruent**

As fixations increased on the alternative image and, later, the target image, there was a steady decrease in fixations on the two foil images in incongruent sentences. For foil 1, fixations decreased from time bin 1 to time bin 2 (*ED* = -.07, *z* = -3.53, *p* < .001) and from time bin 2 to time bin 3 (*ED* = -.11, *z* = -5.40, *p* < .001). Fixations on foil 2 also decreased from time bin 1 to time bin 2 (*ED* = -.08, *z* = -4.28, *p* < .001) and from time bin 2 to time bin 3 (*ED* = -.09, *z* = -4.28, *p* < .001).

Within time bin 1, participants had fewer fixations on the target image than either foil 1 (*ED* = -.05, *z* = -2.70, *p* < .01) or foil 2 (*ED* = -.07, *z* = -3.78, *p* < .001). Participants had more fixations on the alternative image than either foil 1 (*ED* = .07, *z* = 3.56, *p* < .001) or foil 2 (*ED* = .05, *z* = 2.48, *p* < .05). Participants did not differ in their fixations on the two foil images during time bin 1 (*ED* = -.02, *z* = -1.08, *p* > .05).

During time bin 2, there were no differences in fixations on the target image relative to either foil 1 (*ED* = -.00, *z* = -.18, *p* > .05) or foil 2 (*ED* = -.01, *z* = -.49, *p* > .05). There was a significant interaction with age group for the difference between the target image and foil 1 (*ED* = -.04, *z* = -2.01, *p* < .05), but there was no difference in fixations on the target image and foil 1 for either younger adults (*ED* = -.02, *z* = -1.62, *p* > .05) or older adults (*ED* = .02, *z* = 1.24, *p* > .05). Participants had more fixations on the alternative image than either foil 1 (*ED* = .31, *z* = 15.56, *p* < .001) or foil 2 (*ED* = .30, *z* = 15.25, *p* < .001). However, there was a marginally significant interaction with age group for the difference between the alternative image and foil 1 (*ED* = -.04, *z* = -1.93, *p* = .05), indicating that younger adults had more fixations on the alternative image than foil 1 (*ED* = .14, *z* = 10.08, *p* < .001), but this difference was greater for older adults (*ED* = .17, *z* = 11.86, *p* < .001). Fixations on the two foil images did not differ during time bin 2 (*ED* = -.01, *z* = -.30, *p* > .05).

Finally, in time bin 3, participants had more fixations on the target image than either foil 1 (*ED* = .26, *z* = 12.60, *p* < .001) or foil 2 (*ED* = .23, *z* = 11.21, *p* < .001). There was a significant interaction with age group for the difference between the target image and foil 1 (*ED* = .09, *z* = 4.21, *p* < .001), indicating that younger adults had more fixations on the target image than foil 1 (*ED* = .17, *z* = 11.84, *p* < .001), but this difference was smaller for older adults (*ED* = .09, *z* = 5.96, *p* < .001). There was also a significant interaction with age group for the difference between the target image and foil 2 (*ED* = .06, *z* = 2.82, *p* < .01), again indicating that younger adults had more fixations on the target image than foil 2 (*ED* = .15, *z* = 9.89, *p* < .001), but this difference was smaller for older adults (*ED* = .09, *z* = 5.95, *p* < .001). Participants also had more fixations on the alternative image than either foil 1 (*ED* = .46, *z* = 22.16, *p* < .001) or foil 2 (*ED* = .43, *z* = 20.77, *p* < .001). There was a significant interaction with age group for the difference between the alternative image and foil 1 (*ED* = -.17, *z* = -8.23, *p* < .001), indicating that younger adults had more fixations on the alternative image than foil 1 (*ED* = .14, *z* = 9.82, *p* < .001), but this difference was far greater for older adults (*ED* = .31, *z* = 21.57, *p* < .001). There was also a significant interaction with age group for the difference between the alternative image and foil 2 (*ED* = -.20, *z* = -9.61, *p* < .001), again indicating that younger adults had more fixations on the alternative image than foil 2 (*ED* = .12, *z* = 7.86, *p* < .001), but this difference was far greater for older adults (*ED* = .31, *z* = 21.56, *p* < .001). Fixations on the two foil images did not differ during time bin 3 (*ED* = -.01, *z* = -.44, *p* > .05).
